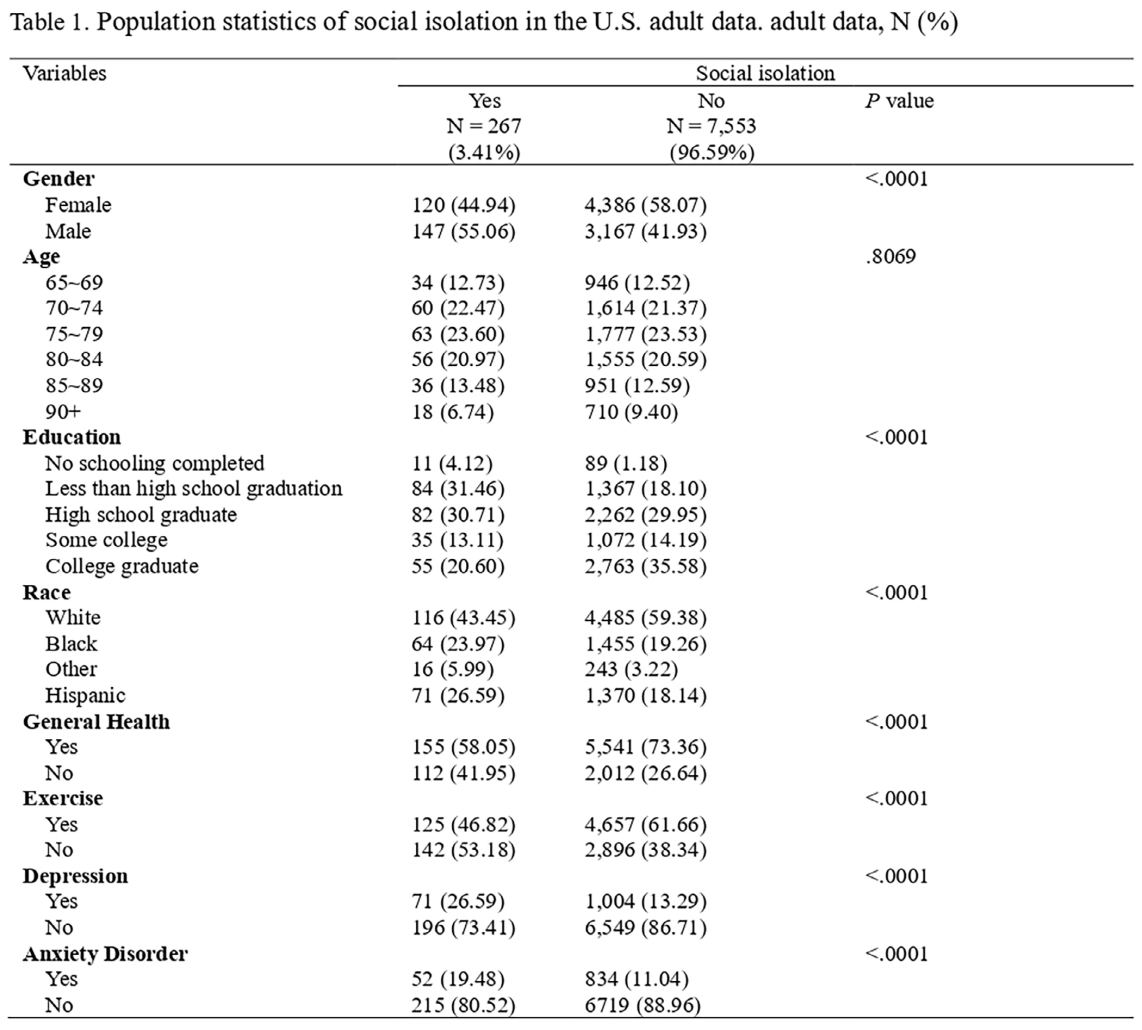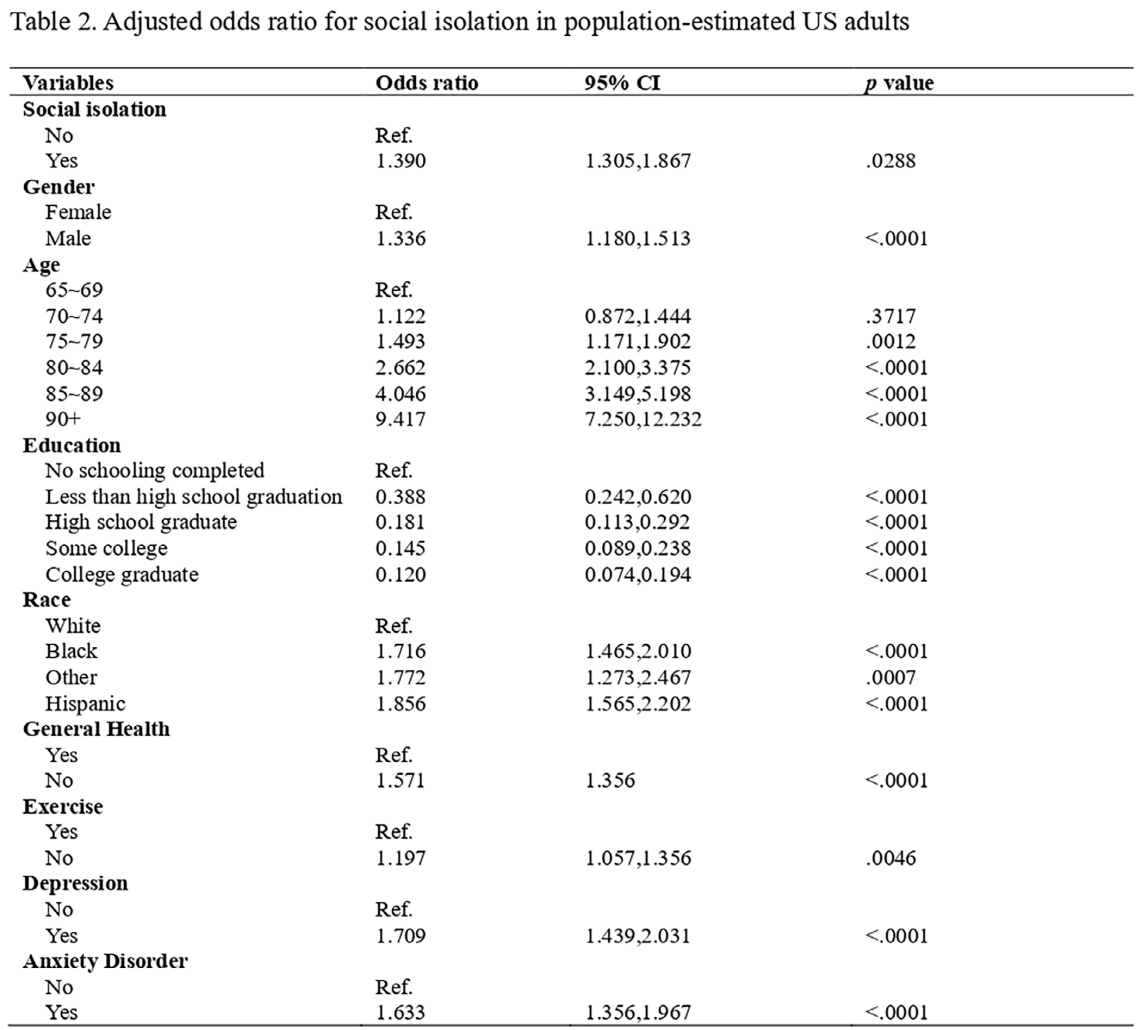# Association Between Social Isolation and Dementia in U.S. Adults

**DOI:** 10.1002/alz70858_098711

**Published:** 2025-12-25

**Authors:** Hokyung Lee, Ickpyo Hong

**Affiliations:** ^1^ Yonsei University, Wonju, Gangwon‐do, Korea, Republic of (South)

## Abstract

**Background:**

Social isolation is a major risk factor for cognitive decline and dementia, emerging as a significant public health issue in an aging society. Previous studies have shown that social isolation is associated with mental health, physical activity, and demographic variables. However, research on the comprehensive impact of these factors on the occurrence of dementia is limited. Therefore, this study aims to analyze how social isolation, along with gender, education level, race/ethnicity, health status, exercise, depression, and anxiety, affects the risk of developing dementia.

**Method:**

This study utilizes data from the 2023 National Health and Aging Trends Study (NHATS) to examine the relationship between social isolation and dementia among American adults. The independent variable was social isolation, and the dependent variable was dementia. Covariates include gender, age, race/ethnicity, educational attainment, exercise, general health status, anxiety, and depression symptoms. Multivariate logistic regression analyses are employed to assess the associations.

**Result:**

According to the demographic results, a total of 267 individuals (3.41%) experienced social isolation, while 7,553 individuals (96.59%) were not socially isolated. Significant differences in social isolation status were observed across several variables, including gender, education level, race, general health status, exercise habits, depression, and anxiety disorders (*p* < .0001). Specifically, individuals who were socially isolated had approximately a 39.0% increased odds of developing dementia (OR = 1.390, 95% CI: 1.305 ‐ 1.867). Those who did not exercise had about a 19.7% higher odds of dementia (OR = 1.197, 95% CI: 1.057 ‐ 1.356, *p* = .0046), and individuals with depression (OR = 1.709, 95% CI: 1.439 ‐ 2.031, *p* < .0001) and anxiety disorders (OR = 1.633, 95% CI: 1.356 ‐ 1.967, *p* < .0001) had approximately 70.9% and 63.3% increased odds of developing dementia, respectively.

**Conclusion:**

This study revealed that social isolation, lack of exercise, depression, and anxiety disorders significantly increase the risk of developing dementia. Specifically, individuals who were socially isolated had a 39% higher odds of developing dementia, and those who did not exercise or had mental health issues also exhibited increased risks of dementia. These findings emphasize the importance of strengthening social support, promoting regular exercise for effective dementia prevention.